# Endovascular pharmacomechanical thrombolysis—a novel treatment for circumaortic left renal vein and inferior vena cava thrombosis in a paediatric patient with relapsing nephrotic syndrome

**DOI:** 10.1259/bjrcr.20170082

**Published:** 2017-11-08

**Authors:** Manraj Kanwal Singh Heran, Tyler Michael Coupal, Janis Dionne

**Affiliations:** ^1^Department of Radiology, British Columbia Children’s Hospital, Vancouver, BC, Canada; ^2^Department of Radiology, Vancouver General Hospital, Vancouver, BC, Canada; ^3^Department of Paediatrics, Division of Nephrology, British Columbia Children’s Hospital, Vancouver, BC, Canada

## Abstract

While venous thromboembolism (VTE) in children with nephrotic syndrome (NS) remains an uncommon clinical entity, it represents one of the disease's most severe and potentially fatal complications. As such, clinicians and radiologists must maintain a high level of suspicion for VTE and low threshold for performing diagnostic imaging studies in children with NS, thereby ensuring prompt diagnosis and early management initiation. Despite the recent advances and development of image-guided endovascular procedures, there remains a marked paucity of literature describing the use of endovascular intervention for the treatment of acute VTE in NS, and a clear consensus on the gold standard for management has yet to be fully elucidated. Moreover, given the relative rarity of this complication in children as opposed to adults, no prior report has been made in which a paediatric patient has undergone endovascular intervention for acute VTE in the setting of NS. This report will outline the use of endovascular pharmacomechanical thrombolysis and thrombectomy as a novel treatment option for acute circumaortic left renal vein and inferior vena cava thrombosis in a paediatric patient with relapsing NS.

## Introduction

Nephrotic syndrome (NS) is characterized by a clinical triad comprised of nephrotic range proteinuria, hypoalbuminemia and peripheral oedema. NS has an incidence of 2–7/100,000 children, with the two most common aetiologies being minimal change NS and focal segmental glomerulosclerosis—both of which can manifest as relapsing disease.^1^ Afflicted children often have increased haematocrit levels and chronic hypoalbuminemia, which manifests clinically as a hypercoagulable state, with a resultant increased risk of venous thromboembolism (VTE).^2,3^ With an estimated incidence of 1.8–5.0%,^1,3^ the risk of VTE in children with NS remains low, but it represents one of the disease’s most severe and potentially fatal complications. Clinicians and radiologists must therefore maintain a high level of suspicion and low threshold for performing diagnostic imaging studies in children with NS, thereby ensuring prompt diagnosis and early management initiation.

Initial clinical management of VTE classically consists of systemic anticoagulation through the use of intravenous heparin or subcutaneous low molecular weight heparin. Other reports have also described the use of systemic thrombolysis, and rarely, surgical thrombectomy can be performed for severe cases that are refractory to medical management. Despite the recent advances and development of image-guided endovascular procedures, there still remains a marked paucity of literature describing the use of endovascular thrombolysis and/or thrombectomy for VTE in NS.^4–9^ As such, a clear consensus on the gold standard for management of acute VTE in NS patients has yet to be fully elucidated. Moreover, given the relative rarity of this complication in children as opposed to adults, no prior report has been made in which a paediatric patient has undergone endovascular intervention for acute VTE in the setting of NS.

We report the use of endovascular pharmacomechanical thrombolysis and thrombectomy as a novel treatment option for acute circumaortic left renal vein and inferior vena cava (IVC) thrombosis in a paediatric patient with relapsing NS.

## Case report

A 16-year-old male presented to the emergency department with acute onset left flank pain and frank haematuria. This patient had a longstanding history of relapsing nephrotic syndrome secondary to minimal change disease, with his most recent relapse occurring 2 weeks prior to this presentation after an attempt to wean his mycophenolate mofetil dosage. His medications on admission were furosemide 60 mg once daily by mouth, prednisone 60 mg once daily by mouth and mycophenolate mofetil 500 mg twice daily by mouth. There was no known family history of VTE or bleeding diatheses. The patient denied recent calf swelling, asymmetry in thigh circumference or leg pain. There was no history of prolonged immobilization, but the patient did report decreased oral intake throughout the week preceding his presentation, secondary to increasing flank pain and associated nausea.

On examination, the abdomen was soft, but the patient was tender to palpation in the left upper quadrant and left flank. Generalized oedema was noted; however, the lower extremities were symmetrical in size with no erythema or tenderness. The patient’s bloodwork showed a haemoglobin of 175 g l^–1^ (reference range: 131–169 g l^–1^), haematocrit 0.5 (reference range: 0.38–0.49), platelets 111 x 10^9^ (reference range: 165–397 x 10^9^) and white blood cell count 20.1 x 10^9^ (reference range: 3.9–10.2 x 10^9^). The patient had a normal electrolyte panel, creatinine of 100 μmol l^–1^ (reference range: 39–103 μmol l^–1^), urea 9.9 mmol l^–1^ (reference range: 2.5–7.1 mmol l^–1^) and albumin 23 mmol l^–1^ (reference range: 37–56 mmol l^–1^). His urinalysis showed 23.84 g l^–1^ protein, a urine protein:creatinine ratio of 1136 g mol^–1^ (reference range: 0–22 g mol^–1^), greater than 100 red blood cells hpf^–1^ (reference range: 0–3/hpf), 10–20 white blood cells hpf^–1^ (reference range: 0–5/hpf), along with the presence of hyaline and granular casts.

**Figure 1.**
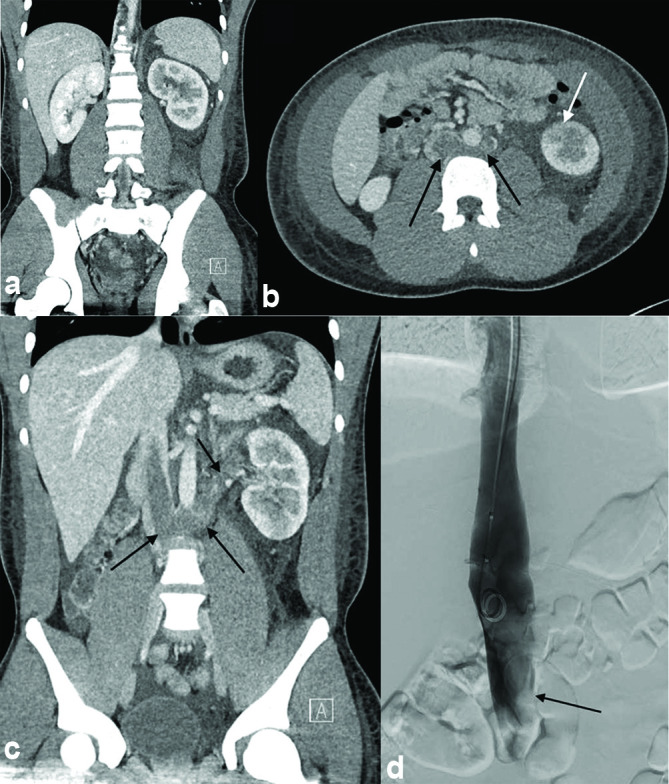
Multiplanar intravenous contrast-enhanced CT of the abdomen and pelvis demonstrates enlargement of the left kidney, moderate left-sided perinephric free fluid (a) and a delayed left nephrogram (white arrow—b). Acute thrombus was noted in a tortuous circumaortic left renal vein, which extends into the inferior vena cava and measures up to 12.0 cm in craniocaudal dimension (black arrows—b, c). Subsequent anteroposterior projection cavogram demonstrates inferior vena cava thrombus, which extends downstream to the approximate level of the right renal vein (black arrow—d).

The initial imaging test ordered was a renal ultrasound to rule out renal calculus or renal vein thrombosis. The Doppler ultrasound showed asymmetric renal volumes with no evidence of hydronephrosis or post-renal obstruction. The renal vasculature, however, could not be adequately assessed. A contrast-enhanced CT scan of the abdomen and pelvis was then ordered, which demonstrated enlargement of the left kidney, moderate perinephric free fluid and a delayed left nephrogram [Figure 1]. Acute complete thrombosis was noted of the circumaortic left renal vein, with extension into the IVC, where it was non-occlusive but measured up to 12.0 cm in craniocaudal dimension [Figure 1]. There was significant intraperitoneal free fluid and a small left-sided pleural effusion. At this time, Paediatric Haematology and Nephrology were consulted for further management of the patient’s acute renal vein and IVC thrombosis.

Paediatric Haematology began anticoagulation with unfractionated heparin; however, given the significant thrombus and the patient’s poor clinical status, interventional radiology was consulted to consider potential intervention. After clinical examination and review of the patient’s CT imaging, a decision was made to attempt endovascular pharmacomechanical thrombolysis and thrombectomy.

The patient was transferred to the angiography suite, intubated and placed under general anaesthetic. The patient's right neck and right groin were prepped and draped followed by ultrasound-assisted right internal jugular vein access. A cavogram was performed in the anteroposterior projection, demonstrating the known IVC thrombus, with tailing thrombus extending downstream to at least the level of the right renal vein [Figure 1].

**Figure 2.**
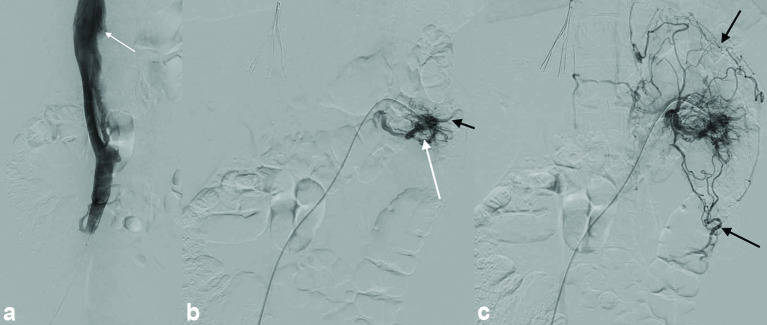
By utilizing the neck access, an inferior vena cava filter was inserted above the level of the inferior vena cava thrombus (white arrow—a), which acted as a safeguard against potential periprocedural thromboembolic events. Hand injection venography via the retroaortic limb of the left renal vein demonstrated complete occlusion of the left renal vein (white arrow—b) with venous return occurring through numerous capsular collaterals extending along the left lateral aspect of the spine (black arrows—b, c).

From the neck access, an Option Elite IVC filter (Argon Medical Devices Inc., Plano, TX) was inserted in a suprarenal position above the level of the IVC thrombus [Figure 2], which acted as a safeguard against potential periprocedural thromboembolic events. Hand injection venography was then performed, demonstrating no patency of the left renal vein with numerous capsular collaterals extending to venous pathways along the left lateral aspect of the spine [Figure 2].

At this point, an Angiojet device (Boston Scientific Corporation, Marlborough, MA) was advanced into the left renal vein, at the level of the hilum. Pulse-spray tissue plasminogen activator (tPA) was administered via the Angiojet device into the left renal vein thrombus as the device was slowly withdrawn back to the expected level of the left renal vein/IVC junction. The catheter was then repositioned within the IVC thrombus and the Angiojet device was advanced to the expected left renal vein/IVC junction, at which point pulse-spray tPA was again performed. The Angiojet device was subsequently repositioned in the upstream portion of the left renal vein. As expected, hand injection performed at this time demonstrated minimal patency of the left renal vein with significant persistent thrombus burden.

**Figure 3.**
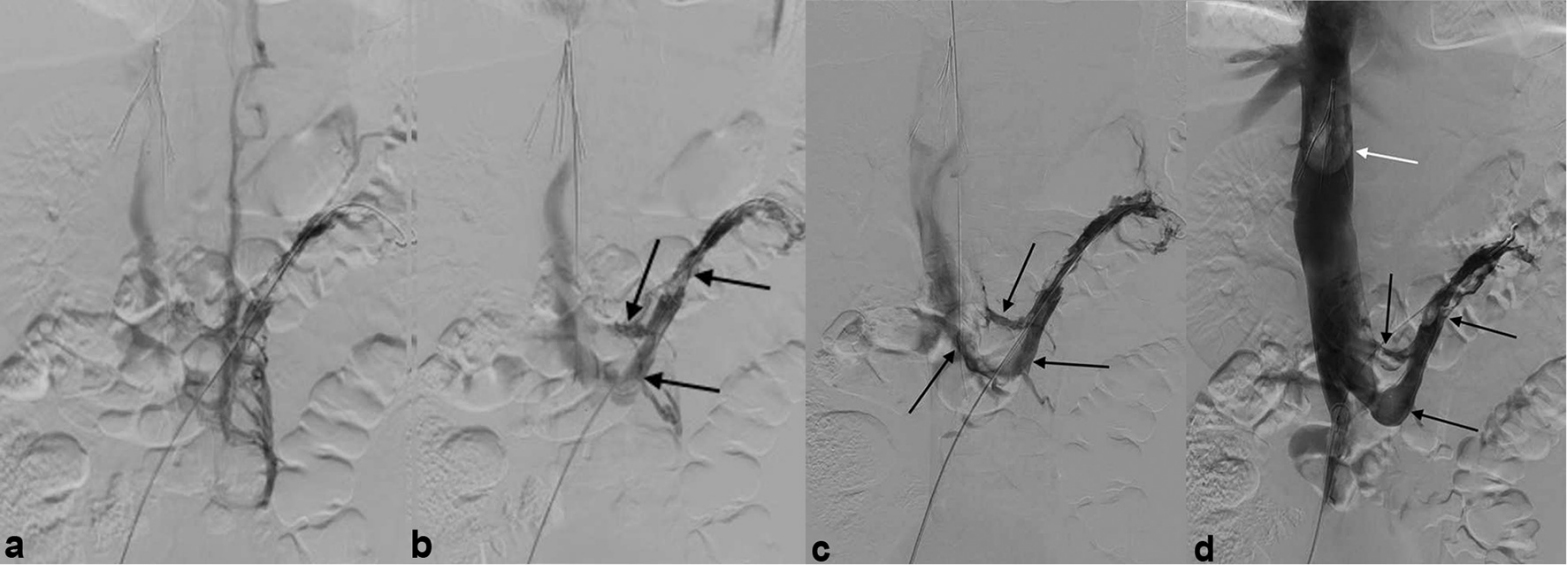
Angiography before (a) and after (b–d) Angiojet pulse spray tissue plasminogen activator thrombolysis and balloon angioplasty demonstrate progressive recanalization of the left renal vein, with opacification now seen in the anterior and posterior limbs of the renal vein and flow extending into the IVC (black arrows—b, c). Pigtail angiography performed following aspiration thrombectomy shows wide patency of the left renal vein (black arrows—d) with a large thrombus having been captured by the in situ IVC filter (white arrow—d), demonstrating the utility and efficacy of prophylactic filter placement when performing such intervention.

After waiting for 20 minutes following tPA administration, aspiration thrombectomy was performed using the Angiojet device for a total of 6 minutes, divided between the left renal vein and IVC thrombus. Angiography demonstrated progressive recanalization of the left renal vein with visualization of the anterior limb of the circumaortic left renal vein and restored flow into the IVC [Figure 3]. Balloon angioplasty of the retroaortic limb of the left renal vein was then performed to macerate all remaining thrombus and increase the surface area of thrombus available for exposure to blood flow, which further improved patency of the left renal vein [Figure 3]. Pigtail angiography performed following IVC aspiration thrombectomy showed wide patency, with a large thrombus having been captured by the recently placed IVC filter [Figure 3]. Repeat angiogram demonstrated good flow in the left renal vein [Figure 3], and as such, no further recanalization intervention was pursued.

Finally, mechanical aspiration thrombectomy of thrombus captured by the IVC filter was performed utilizing the Penumbra aspiration system (Penumbra Inc., Alameda, CA). The patient tolerated the procedure well with no immediate complications and the patient was continued on systemic anticoagulation. The IVC filter was removed without complications 4 days post-intervention, and follow-up renal ultrasound at 6 days post-intervention showed the IVC and left renal vein to be patent without evidence of residual or recurrent thrombus.

## Discussion

VTE in paediatric patients with NS, while uncommon, remains a serious complication with the potential for significant associated morbidity or mortality. The mechanisms in which VTE develops are two-fold: first, increased urinary excretion of endogenous anticoagulant proteins (*i.e.* protein C, protein S, antithrombin III, etc.), and second, increased synthesis of prothrombotic factors (*i.e.* fibrinogen, von Willebrand factor, factor V, factor VIII, etc.).^10^ Additional risk factors such as hypovolemia, diuretic therapy and the presence of indwelling catheters have also been shown to further exacerbate the risk of VTE in NS patients.^2,11,12^

The diagnosis of VTE, and specifically renal vein or IVC thrombosis, can be made through the use of multiple diagnostic imaging modalities, including ultrasound, MRI, contrast-enhanced CT and formal catheter venography. While venography is still considered the gold standard for diagnosis, recent literature has shifted towards strongly advocating for the use of non-invasive imaging modalities such as ultrasound, CT or MRI. In paediatric populations, Doppler ultrasound is often used as an initial diagnostic tool given its ability to perform real-time haemodynamic assessment in the absence of ionizing radiation exposure. As demonstrated in this case report, however, ultrasound’s efficacy is variable and can be highly operator dependent, and cross-sectional imaging may be required for definitive diagnosis and to delineate disease extent. Although CT does impart ionizing radiation, it can be performed rapidly, which is particularly important in the paediatric setting where this may allow for examinations to be done without the need for anaesthetic sedation. Although MRI would be an ideal test in paediatric populations given the absence of ionizing radiation, there is a high likelihood of requiring conscious sedation or general anaesthesia due to the significantly longer image acquisition time. For these reasons, contrast-enhanced CT is often the best secondary imaging study in the setting of an inconclusive ultrasound.

We describe a rare case of a paediatric patient with relapsing NS successfully treated with endovascular pharmacomechanical thrombolysis and thrombectomy after presenting to the emergency department with acute large volume circumaortic left renal vein and IVC thrombus. While a small number of reports in the current literature have described similar interventional approaches,^4–9^ this is the first report of a paediatric patient with NS undergoing endovascular pharmacomechanical intervention. This case report also demonstrates the efficacy of placing a prophylactic IVC filter to safeguard against periprocedural thromboembolic events when treating large volume thrombus with pharmacomechanical thrombolysis. In light of the findings in this case, it is our hope to have demonstrated the benefits of prophylactic IVC filter placement and to advocate for its consideration among interventionalists when planning such procedures. In conclusion, this case report demonstrates that similar endovascular procedures can be safely performed in paediatric NS patient cohorts presenting with acute VTE, particularly in the setting of extensive thrombus burden or the presence of haemodynamically significant thrombus with clinical deterioration.

## Learning Points

1. While the clinical management of VTE classically consists of systemic anticoagulation through the use of intravenous heparin or subcutaneous low molecular weight heparin, pharmacomechanical thrombectomy is a safe and efficacious treatment option for paediatric patients with nephrotic syndrome presenting with acute VTE.
2. Endovascular intervention for the treatment of acute VTE should particularly be advocated for in the setting of paediatric patients presenting with extensive thrombus burden or the presence of haemodynamically significant thrombus with clinical deterioration.
3. Placement of a prophylactic IVC filter can reduce associated morbidity and mortality by safeguarding against periprocedural thromboembolic events when performing such procedures and can be one of the many considerations for interventionalists when planning the treatment of large volume thrombus via pharmacomechanical thrombolysis.

## Consent

Written informed consent was obtained from the patient for publication of this case report, including accompanying images.
